# Cancer-associated financial burden in German head and neck cancer patients

**DOI:** 10.3389/fonc.2024.1329242

**Published:** 2024-01-26

**Authors:** Jonas Rast, Veit Zebralla, Andreas Dietz, Gunnar Wichmann, Susanne Wiegand

**Affiliations:** Department of Otorhinolaryngology, Head and Neck Surgery, University Hospital Leipzig, Leipzig, Germany

**Keywords:** head and neck cancer, financial burden, financial toxicity, out-of-pocket costs, income loss, supportive care, cancer survivorship

## Abstract

**Background:**

The financial toxicity of cancer causes higher morbidity and mortality. As the financial burden due to head and neck cancer (HNC) in European healthcare systems with legally established compulsory health insurance is still poorly understood, we set up an investigation to assess the financial impact of HNC.

**Methods:**

Between August 2022 and March 2023, HNC consecutive patients (*n* = 209) attending the cancer aftercare program of a university hospital in an outpatient setting were surveyed utilizing self-administered questionnaires about their socioeconomic situation, income loss, and out-of-pocket payments (OOPPs).

**Results:**

The majority of HNC patients (*n* = 119, 59.5%) reported significant financial burden as a consequence of OOPP (*n* = 100, 50.0%) and/or income loss (*n* = 51, 25.5%). HNC patients reporting financial burden due to OOPP had on average 1,716 € per year costs related to their disease, whereas patients reporting an income loss had a mean monthly income loss of 620.53 €. Advanced UICC (7th edition, 2017) stage, T3 or T4 category, and larynx/hypopharynx cancer are significant predictors of financial burden.

**Conclusion:**

HNC survivors suffer from significant financial burden after HNC treatment, even in Germany with a healthcare system with statutory health insurance. The findings from this study offer valuable insights for healthcare professionals and policymakers, helping them acknowledge the economic impact of HNC.

## Introduction

Today, we observe enormous progress in cancer treatment with increasingly new options to potentially improve patient outcomes (1). However, these new achievements in modern medicine are associated with a substantial burden for almost every country of the Organization for Economic Co-operation and Development (OECD) over the last years due to rising health expenditures (2). As a result, a portion of the costs has shifted to the population in the form of higher deductibles, copayments, and tiered drug formularies, creating the risk wherein certain subgroups are affected unequally. Cancer survivors may face financial hardship due to excessive financial strain resulting from improved survival, escalated utilization of expensive innovative cancer therapies, and copayments in the current healthcare system (3). Research about the described effect has started with a focus on the US healthcare system and introduced a new terminology—”financial toxicity” (4, 5). Although this phenomenon is still not fully understood, it is defined by the consequences of objective financial burden from direct and indirect treatment costs and the subjectively recognized distress arising from these costs (6). To measure the individual financial toxicity, a multifactorial etiology has to be considered, with research suggesting parameters including out‐of‐pocket payments (OOPPs), both absolute and as percentage of income, and loss of income due to missed work, reduction in assets, and return to work (or incapability to keep on working). Moreover, payments for healthcare including follow-up diagnostics, costs of drugs, additional treatments, and supportive needs (such as travel) can further contribute. Surveys on distress or quality of life (QoL) provide knowledge of psychological consequences (2, 4, 7). Studies showed that research was rarely conducted in none third-party payer healthcare systems like Germany, and consequently, there are only a few studies from Europe (8). Evidence from countries with legally established compulsory health insurance is still lacking and hence necessitates future research, especially in underserved groups of patients, e.g., patients with head and neck cancer (HNC).

The demographic and clinical features of HNC patients, and head and neck squamous cell carcinoma (HNSCC) in particular, may make them especially susceptible to financial hardship. Patients with HNSCC are more often underprivileged, rather poor, less well-educated, have mostly an impaired general health status and nutritional status, and even after successful (curative) treatment return to work less frequently than survivors of other cancers (9–11). Psychological disorders including anxiety and depression, particularly in patients with tumor and treatment-related disfigurement, are affiliated with HNC patients undergoing ablative surgery (12, 13). However, addiction to alcohol or nicotine abuse is more frequent in HNSCC patients, and as these are linked to a multitude of comorbidities, these lifestyle-associated risk factors increase the financial burden further. It could be demonstrated in several studies that being a current smoker or drinker is associated with financial distress (14–17). Among HNSCC patients, associations have been shown between financial burden and decreased quality of life, worse disease outcomes, lower satisfaction with cancer care, and deprived treatment adherence, leading to increased costs and hence even higher risk for financial toxicity (18, 19). Despite these well-known differences between patients suffering from HNC and other cancer patients, tailored tools addressing the potential needs of this underserved patient population appear to be inexistent, probably related to missing knowledge about particular needs. The latter may also be linked to so far unknown financial burden even in healthcare systems with statutory health insurance. To fill this knowledge gap, we conducted a study to determine the financial burden caused by head and neck cancer.

## Methods

### Statistical considerations and sample size

We prospectively designed a study to investigate the socioeconomic impact of HNSCC on the financial burden of patients. The formula used for sample size calculation along with error margin *ϵ* was as follows:


(1) $$n={\left(Z_{\text{alpha}}/2\right)}^{2}\times P\times (1-P)/\epsilon^{2}$$

where *Z*_alpha_/2 is the level of *u* for significance at 5% = 1.96, *P* = prevalence of toxic effects on income expected (*i.e.*, 25%, or 0.25), and *ϵ* = desired error margin (i.e., 10%, or 0.10).

So, according to equation (1), *n* = (1.96 × 1.96 × 0.25 × (1 − 0.25))/0.10 × 0.10 = 72.03 or, *n* = 73 cases at minimum.

Thus, expecting a dropout of approximately 25% caused by incomplete questionnaires, a sample size of *n* = 98 patients would be required for the study, and *n* = 196 patients in total should be invited to answer the questionnaires.

The study was approved by the Ethics Committee of the Medical Faculty of the University Leipzig (vote 289722-ek) and performed in accordance with the ethical standards as laid down in the 1964 Declaration of Helsinki and its later amendments. All participants provided written informed consent.

### Study design and participants

This prospective survey study included consenting German-speaking adult patients diagnosed and treated in the Department of Otolaryngology, Head and Neck Surgery who presented to the HNSCC aftercare program from August 2022 to March 2023. All patients were included in the aftercare program 2 to 3 months after completion of active HNSCC treatment. On a weekly basis, *n* = 209 patients aged 18 years and older with HNC were consecutively invited to participate until reaching the prespecified number of informative cases (*n* > 196) required. The statistical analysis regarding financial burden was performed on *n* = 200 patients because *n* = 9 patients were excluded due to missing or inconsistent information linked to financial burden.

### Survey instrument

We conducted this study by handing out a questionnaire when the patients attended their aftercare appointment. The questionnaire, which was inspired by the patient survey by Mehlis et al. (20), contains 25 questions on cancer-related out-of-pocket costs, monthly household income and income loss, employment status, and behavioral consequences, as well as demographic data to obtain both, direct and indirect cancer-related expenses, and patients’ individual coping strategy. All participating patients had sufficient knowledge of the German language in order to complete the questionnaire on their own, with the help of an assistant so that any arising questions could be answered on-site. The survey was completed once by each patient.

Additionally, clinical and disease-related parameters were extracted from each patient’s records. The following patient data, which could influence financial burden, were evaluated: sex, age, tumor site and stage, recurrence, and treatment regimens.

### Statistical analysis

Case number and frequency of patient characteristics were compared using contingency tables and Pearson’s chi-squared (*χ*^2^) tests and cardinal-metric covariates applying Student’s *t*-tests for homo- or heteroscedastic comparisons, as appropriate. For defining income and expenditure categories, we adhered to recently published intervals (20). By considering only payments or costs appearing on a regular basis (i.e., not considering one-time payments), financial burden was defined as the sum of loss in yearly income and expenditure exceeding the null (0). Patients who were already retired at the time of diagnosis were considered as without loss in income, whereas patients unemployed or with low income at the time of diagnosis and thereafter receiving pension (no matter the source) may have been categorized as without loss in income, depending on the individual’s difference of both self-reported values. Besides considering the nominal category of the interval, centered (mean) values for the particular interval were used in the calculations. Whenever a patient stated to have “any loss in income” despite no difference in net income before diagnosis and after therapy according to stated categories, or, vice versa, having stated “no loss in income” despite belonging to differing income categories before and after therapy, we classified the patient as a patient having financial burden.

All statistical analyses were performed using SPSS version 29 (IBM Cooperation, Armonk, NY, USA) and included logistic regression for multivariate analyses. To this end, we used the stepwise forward option for likelihood ratio-based extraction of independent predictors for financial burden among covariates with *p >*0.2 in the univariate analyses.

## Results

The study cohort, eligible for statistical analysis, consisted of 200 patients. A total of 73.5% of these patients were men, and the mean age was 64.2 years. Demographic parameters and characteristics of the tumor localization, histology, tumor classification, and performed therapy modality are summarized in **Table 1**.

**Table 1:** Cancer characteristics of the study participants.

Variables	Total, *n* = 200 No. (%)	Financial burden group, *n* = 119 No. (%)	Non-financial burden group, *n* = 81 No. (%)	OR (95% CI)	*p*-value
Sex					0.6325
Male	147 (73.5)	86 (43.0)	61 (30.5)	1 (0.629–1.59)	
Female	53 (26.5)	33 (16.5)	20 (10.0)	1.17 (0.614–2.231)	
Age group					0.4928
18–50 years	14 (7.0)	9 (4.5)	5 (2.5)	1 (0.213–4.693)	
51–60 years	50 (25.0)	32 (16.0)	18 (9.0)	1.013 (0.294–3.486)	
61–70 years	81 (40.5)	50 (25.0)	31 (15.5)	1.116 (0.342–3.637)	
>70 years	55 (27.5)	28 (14)	27 (13.5)	1.736 (0.515–5.846)	
Marital status					0.4463
Single	27 (13.5)	17 (14.3)	10 (12.3)	1 (0.331–3.018)	
Married	121 (60.5)	70 (58.8)	51 (63.0)	0.807 (0.342–1.909)	
Widowed	16 (8.0)	8 (6.7)	8 (9.9)	0.588 (0.168–2.06)	
Living with partner	12 (6.0)	10 (8.4)	2 (2.5)	2.941 (0.533–16.219)	
Other	24 (12.0)	14 (11.8)	10 (12.3)	0.824 (0.267–2.54)	
Highest school degree					0.2542
General elementary education	77 (38.3)	46 (38.7)	31 (38.3)	1 (0.525–1.904)	
Intermediate vocational qualification or intermediate general qualification	61 (34.6)	33 (27.7)	28 (34.6)	0.794 (0.403–1.566)	
General maturity certificate	56 (22.2)	38 (31.9)	18 (22.2)	1.423 (0.691–2.93)	
Other	6 (3.0)	2 (1.7)	4 (4.9)	0.337 (0.058–1.954)	
Insurance status					
Private insured	8 (4.1)	6 (5.0)	2 (2.5)	1 (0.104–9.614)	0.4366
Statutory insured	186 (94.4)	110 (92.4)	76 (93.8)	0.482 (0.095–2.455)	
Other incl. free of charge coinsured	6 (3.0)	3 (2.5)	3 (3.7)	0.167 (0.009–2.984)	
p16+ oropharynx cancer (OPSCC) vs. other					0.5899
Other	144 (72.0)	84 (42.0)	60 (30.0)	1 (0.626–1.598)	
p16+ OPSCC	56 (28.0)	35 (17.5)	21 (10.5)	1.19 (0.631–2.245)	
Surgery only					0.0558
No	150 (75.0)	95 (47.5)	55 (27.5)	1 (0.625–1.599)	
Yes	50 (25.0)	24 (12.0)	26 (13.0)	1.871 (0.98–3.572)	
Surgery and adjuvant radio(chemo)therapy					0.1367
No	91 (45.5)	49 (24.5)	42 (21.0)	1 (0.558–1.791)	
Yes	109 (54.5)	70 (35.0)	39 (19.5)	1.538 (0.871–2.716)	
Definitive radio- or radiochemotherapy					0.5963
No	167 (83.5)	98 (49.0)	69 (34.5)	1 (0.647–1.546)	
Yes	33 (16.5)	21 (10.5)	12 (6.0)	1.232 (0.569–2.67)	
N3 vs. other					0.3649
Other	180 (90.0)	72 (36.0)	108 (54.0)	1 (0.656–1.525)	
N3 7th ed.	7 (3.5)	3 (1.5)	4 (2.0)	2 (0.435–9.203)	
T4 vs. other					**0.0120**
Other	157 (78.5)	87 (43.5)	70 (35.0)	1 (0.641–1.561)	
T4 7th ed.	30 (15.0)	24 (12.0)	6 (3.0)	**3.218 (1.247**–**8.308)**	
T3/T4 vs. other					**0.0008**
Other	128 (64.0)	65 (32.5)	63 (31.5)	1 (0.613–1.632)	
T3 or T4 7th ed.	72 (36.0)	54 (27.0)	18 (9.0)	**2.908 (1.539**–**5.493)**	
M stage					0.7001
M0	183 (91.5)	109 (54.5)	74 (37.0)	1 (0.659–1518)	
M1	4 (2.0)	2 (1.0)	2 (1.0)	1.473 (0.203–10.691)	
Extranodular extension (ENE)					0.3918
N/A. N0	75 (37.5)	41 (20.5)	34 (17.0)	1 (0.526–1.902)	
ENE	41 (20.5)	24 (12.0)	17 (8.5)	1.171 (0.542–2.528)	
No ENE	62 (31.0)	41 (20.5)	21 (10.5)	1.619 (0.808–3.245)	
Tumor recurrence					0.0716
No	172 (86.0)	98 (49.0)	74 (37.0)	1 (0.653–1.532)	
Yes	28 (14.0)	21 (10.5)	7 (3.5)	2.265 (0.914–5.612)	
Larynx/hypopharynx vs. other					**0.0376**
Other localization	150 (75.0)	83 (41.5)	67 (33.5)	1 (0.634–1.577)	
Larynx/hypopharynx	50 (25.0)	36 (18.0)	14 (7.0)	**2.076 (1.035**–**4.164)**	
Advanced vs. early cancer					**0.0186**
Early	56 (28.0)	26 (13.0)	30 (15.0)	1 (0.476–2.102)	
Advanced	131 (65.5)	85 (42.5)	46 (23.0)	**2.132 (1.129–4.027)**	
Advanced larynx/hypopharynx and early larynx/hypopharynx vs. other					**8.7510^−4^**
Early other	36 (18.0)	17 (8.5)	19 (9.5)	1 (0.396**–**2.523)	
Advanced other	103 (51.5)	60 (30.0)	43 (21.5)	1.56 (0.727**–**3.343)	
Early larynx/hypopharynx	20 (10.0)	9 (4.5)	11 (5.5)	0.914 (0.305**–**2.74)	
Advanced larynx/hypopharynx	28 (14.0)	25 (12.5)	3 (1.5)	**9.314 (2.379–36.458)**	

Significant p-values are in bold.

The majority of our cohort faces financial burden (59.5%), and there was no difference with respect to age score, sex, or type of insurance (SHI vs. private). The financial burden group consisted quantitatively more of OOPP than income loss (50.0% vs. 25.5%). Patients with financial burden, who reported OOPP, had on average 1,716 € per year additional costs related to their disease. The most common causes of OOPP were deductibles (*n* = 74) and transportation (*n* = 67), as shown in **Table 2**. Patients with financial burden, who received surgery followed by adjuvant therapy (postoperative radio- or radiochemotherapy) had higher annual mean OOPP than patients with either surgery alone, primary radiochemotherapy (PRCT), or palliative radio(chemo)therapy (1,946 € per year vs. 1,263 € per year). When comparing patients with financial burden categorized into advanced and early-stage tumors according to the UICC 2017, a similar result was found for the extent of OOPP (1,935 € per year in advanced stage vs. 1,255 € per year in early-stage tumors).

**Table 2:** Prevalence and extent of out-of-pocket payments (OOPPs).

Variables	Total *n* = 200 No. (%)	Financial burden group, *n* = 119 No. (%)	Non-financial burden group, *n* = 81 No. (%)	OR (95% CI)	*p*-value
Do you have due to your cancer disease higher out-of-pocket payments? (z.B.: taxi fares, additional charges for pharmaceuticals, treatments)					**1.91 · 10^−23^**
No	82 (41.0)	16 (8.0)	66 (33)	1 (0.462–2.165)	
Yes	113 (56.5)	102 (51)	11 (5.5)	38.25 (16.716–87.524)	
Invalid/unkown	5 (2.5)				
How high are your out-of-pocket payments?					**2.39 · 10^−23^**
N/A	82 (41.0)	16 (8.0)	66 (33.0)	1 (0.467–2.143)	
1 to 99 €	50 (25.0)	47 (23.5)	3 (1.5)	54.697 (16.277–183.802)	
100 to 200 €	41 (20.5)	39 (19.5)	2 (1.0)	63.679 (15.904–254.961)	
201 to 500 €	10 (5.0)	10 (5.0)	0 (0)	84.636 (4.714–1519.613)	
501 to 800 €	4 (2.0)	4 (2.0)	0 (0)	36.273 (1.859–707.809)	
Invalid/unkown	13 (6.5)				
Out-of-pocket payments due to additional charges					**3.56 · 10^−22^**
N/A	82 (41.0)	16 (8.0)	66 (33.0)	1 (0.462–2.165)	
No	38 (19.0)	34 (17.0)	4 (2.0)	35.063 (10.869–113.106)	
Yes	74 (37.0)	67 (33.5)	7 (3.5)	39.482 (15.254–102.194)	
Invalid/unkown	6 (3.0)				
Out-of-pocket payments due to travel expenses					**3.07 · 10^−22^**
N/A	82 (41.0)	16 (8.0)	66 (33.0)	1 (0.462–2.165)	
No	45 (22.5)	42 (21.0)	3 (1.5)	57.75 (15.859–210.298)	
Yes	67 (33.5)	59 (29.5)	8 (4.0)	30.422 (12.142–76.222)	
Invalid/unkown	6 (3.0)				
Out-of-pocket payments due to household help					**3.57 · 10^−22^**
N/A	82 (41.0)	16 (8.0)	66 (33.0)	1 (0.462–2.165)	
No	103 (51.5)	93 (46.5)	10 (5.0)	38.363 (16.384–89.822)	
Yes	9 (4.5)	8 (4.0)	1 (0.5)	33 (3.846–283.143)	
Invalid/unkown	6 (3.0)				
Out-of-pocket payments due to treatments and drugs uncovered by your insurance					**3.58 · 10^−22^**
N/A	82 (41.0)	16 (8.0)	66 (33.0)	1 (0.462–2.165)	
No	70 (35.0)	63 (31.5)	7 (3.5)	37.125 (14.316–96.277)	
Yes	42 (21.0)	38 (19.0)	4 (2.0)	39.188 (12.21–125.771)	
Invalid/unkown	6 (3.0)				

Significant p-values are in bold.

Analyzing all *n* = 51 patients who reported income loss (**Table 3**) due to their tumor disease, the mean monthly income loss was 620.53 €. Patients who received surgery and adjuvant therapy even reported approximately a mean monthly income loss of 703.16 €, and patients with advanced stage according to the UICC 2017 had approximately a mean monthly income loss of 662.12 €, leading to a yearly income loss of 7,446 € and 8,438 €, respectively.

**Table 3:** Explanatory variables and prevalence of income loss.

Variables	Total, *n* = 200 No. (%)	Financial burden group, *n* = 119 No. (%)	Non-financial burden group, *n* = 81 No. (%)	OR (95% CI)	*p*-value
Employment status at the time of diagnosis					**0.0025**
Employment	81 (40.5)	59 (29.5)	22 (11.0)	1 (0.5–1.999)	
Self-employment	8 (4.0)	3 (1.5)	5 (2.5)	4.47 (0.985–20.29)	
Civil servant	2 (1.0)	1 (0.5)	1 (0.5)	2.682 (0.161–44.758)	
Part-time employment	7 (3.5)	3 (1.5)	4 (2.0)	3.576 (0.74–17.274)	
No employment	29 (14.5)	9 (4.5)	20 (10.0)	5.96 (2.359–15.054)	
Retired	70 (35.0)	43 (21.5)	27 (13.5)	1.684 (0.848–3.346)	
Invalid/unkown	3 (1.5)				
Net income before tumor diagnosis					**0.0189**
Retired	70 (35.0)	43 (21.5)	27 (13.5)	1 (0.509–1.965)	
No own income	8 (4.0)	1 (0.5)	7 (3.5)	7.909 (1.285–48.665)	
1–500 €	8 (4.0)	3 (1.5)	5 (2.5)	2.486 (0.599–10.312)	
501–1,000 €	12 (6.0)	5 (2.5)	7 (3.5)	2.157 (0.65–7.158)	
1,001–1,500 €	25 (12.5)	18 (9.0)	7 (3.5)	0.641 (0.242–1.698)	
1,501–2,000 €	22 (11.0)	14 (7.0)	8 (4.0)	0.927 (0.351–2.451)	
2,001–2,500 €	19 (9.5)	14 (7.0)	5 (2.5)	0.6 (0.201–1.787)	
2,501–3,000 €	9 (4.5)	5 (2.5)	4 (2.0)	1.294 (0.341–4.914)	
3,001–3,500 €	3 (1.5)	0 (0)	3 (1.5)	11.073 (0.55–222.728)	
>3,500 €	6 (3.0)	5 (2.5)	1 (0.5)	0.431 (0.067–2.792)	
Invalid/unkown	18 (9.0)				
Current employment status					0.79423
Retired before cancer diagnosis	70 (35.0)	43 (21.5)	27 (13.5)	1 (0.509 - 1.965)	
Employment	45 (22.5)	28 (14.0)	17 (8.5)	0.971 (0.453 - 2.083)	
Self-employment	5 (2.5)	2 (1.0)	3 (1.5)	2.215 (0.408 - 12.024)	
Civil servant	1 (0.5)	0 (0)	1 (0.5)	4.745 (0.187 - 120.696)	
Part-time employment	4 (2.0)	3 (1.5)	1 (0.5)	0.678 (0.094 - 4.868)	
No employment	22 (11.0)	13 (6.5)	9 (4.5)	1.113 (0.427 - 2.9)	
Retired after cancer diagnosis	48 (24.0)	27 (13.5)	21 (10.5)	1.237 (0.591 - 2.59)	
Invalid/unkown	5 (2.5)				
Are you absent due to illness?					**0.0175**
No	99 (49.5)	52 (26.0)	47 (23.5)	1 (0.572 - 1.747)	
Yes	21 (10.5)	18 (9.0)	3 (1.5)	5.423 (1.501 - 19.59)	
Retired	70 (35)	43 (21.5)	27 (13.5)	1.439 (0.773 - 2.682)	
Invalid/unkown	10 (5.0)				
Any loss of income due to your tumor diagnosis					**6.50 · 10^-14^**
No	74 (37.0)	21 (10.5)	53 (26.5)	1 (0.489 - 2.044)	
Yes	51 (25.5)	50 (25.0)	1 (0.5)	126.19 (16.359 - 973.397)	
Retired	70 (35)	43 (21.5)	27 (13.5)	4.019 (2 - 8.077)	
Invalid/unkown	5 (2.5)				
Loss in income per month					**0.0010**
Retired	70 (35.0)	43 (21.5)	27 (13.5)	1 (0.509 - 1.965)	
1 to 99 €	2 (1.0)	2 (1.0)	0 (0)	3.161 (0.146 - 68.347)	
100 to 200 €	4 (2.0)	4 (2.0)	0 (0)	1.897 (0.281 - 12.79)	
201 to 500 €	19 (9.5)	19 (9.5)	0 (0)	24.655 (1.43 - 425.166)	
501 to 800 €	8 (4.0)	8 (4.0)	0 (0)	10.747 (0.596 - 193.747)	
801 to 1,200 €	7 (3.5)	7 (3.5)	0 (0)	9.483 (0.521 - 172.74)	
>1,200 €	8 (4.0)	8 (4.0)	0 (0)	10.747 (0.596 - 193.747)	
Invalid/unkown	82 (41.0)				

Significant p-values are in bold.

**Figure 1:**
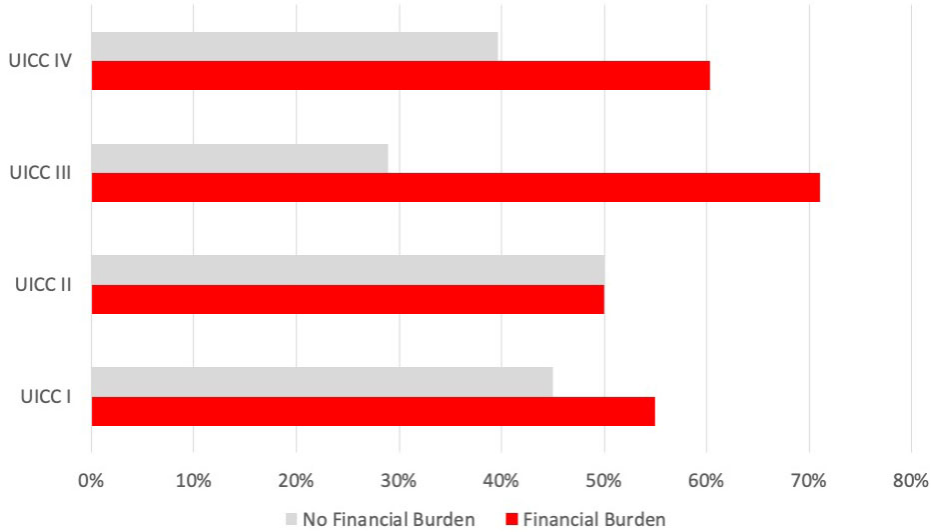
Prevalence of financial burden associated with local tumor size (T category) in all HNC patients (*n* = 200). Bar indicating the number of affected patients with financial burden (in percent) for each T category (7th edition, 2017).

For the subgroup of *n* = 85 patients with reported income loss and OOPP categorized into advanced and early stage according to the UICC 2017, the mean monthly income loss was 454.08 € compared with 248.08 €. Financial burden, in general, was significantly higher in patients with advanced HNSCC (*p* < 0.019) (**Figure 1**); in particular, patients with local advanced larynx or hypopharynx tumor (laryngeal and hypopharyngeal squamous cell carcinoma, LHSCC) have a very high risk (OR = 9.314, 95% CI 2.379–36.458; *p* < 0.001) for financial burden.

**Figure 2:**
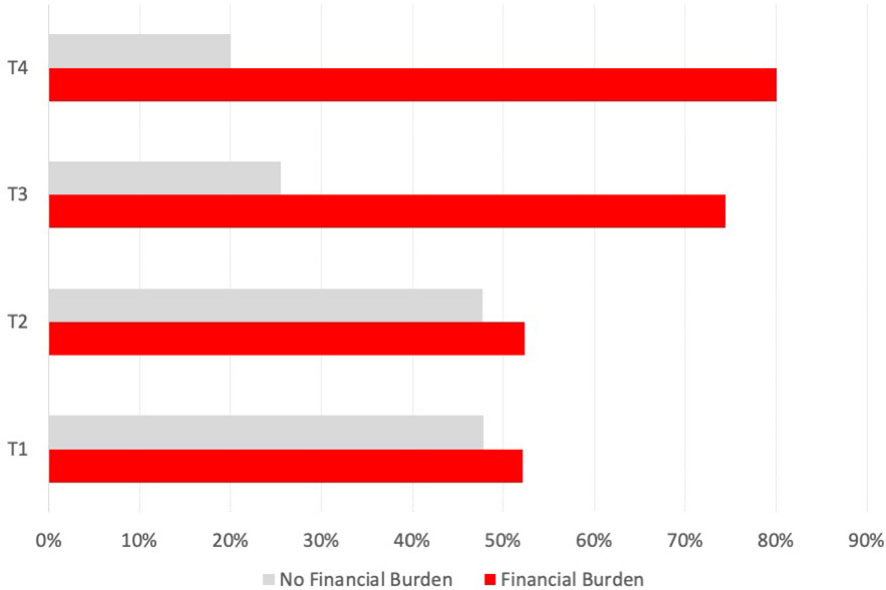
Prevalence of financial burden associated with UICC stage in all HNC patients. Bar indicating the number of affected patients with financial burden (in percent) for each UICC stage (7th edition, 2017).

There was a significant difference between T3 and T4 patients (according to the 7th TNM 2017 edition) having more financial burden (**Figure 2**) compared with patients having a lower T category, which is accompanied by an OR of 2.908 (95% CI 1.539–5.493; *p* < 0.001). There was no significant correlation between the presence of financial burden and N and M categories. However, analyzing all *n* = 24 patients with reported income loss and OOPP (12.0%), the mean monthly financial burden in the group with extranodal extension was higher compared with the group without extranodal extension (570.79 € vs. 357.90 €).

According to the model achieving the highest significance, the sensitivity to detect patients with financial burden was 85.4%, and the prediction accuracy was 60.8%. While patients suffering from advanced stage HNSCC had a 2.7-fold risk to experience financial burden (OR 2.708, 95% CI 1.364–5.378; *p* = 0.0044, bootstrap *p*^#^ = 0.006), this risk was further increased in patients with LHSCC. LHSCC patients had additionally a 2.5-fold increased risk to experience financial burden (OR 2.535, 95% CI 1.195–5.377); *p* = 0.0153, bootstrap *p*^#^ = 0.009).

In the financial burden group, a higher rate of cancer recurrences and p16-positive oropharyngeal SCC could be observed, which, however, due to rather low numbers of patients, failed to reach significance (*p* > 0.1).

The current self-reported employment status did not differ significantly between patients with and without financial burden. Nevertheless, slightly more patients in the group facing financial burden were unemployed after therapy than before diagnosis (**Table 3**). We detected a significant correlation between receiving sick pay, which is paid in Germany from the health insurance company after the employer’s payment of wages during illness expires, and the presence of disease-related financial burden (OR = 20.15, 95% CI 1.179–344.3; *p* < 0.0024).

Patients without a high school degree facing financial burden (*n* = 88) had higher mean monthly OOPP and higher income loss compared with patients with a high school degree (*n* = 29) (407.93 € vs. 339.62 €). A similar trend could be observed when categorizing patients with financial burden into “no college degree” and “college degree” (393.46 € vs. 293.96 €).

Despite not reaching statistical significance, only patients with financial burden stated that they had difficulties in receiving or applying for social services (*n* = 6).

Both OOPP and income loss are self-reported reasons for patients behind cutting their expenses for leisure, food and nutrition, living and household and medical treatments, and other services (all with *p* < 0.0001). Patients with financial burden have a significant risk to experience deterioration in their quality of life (OR = 5.144, 95% CI 2.722–9.721; *p* < 0.0001).

## Discussion

HNSCC survivors are vulnerable to financial hardship (21). The present study aimed to investigate the prevalence of financial burden and socioeconomic impact of HNSCC on patients in Germany and their direct and indirect cancer-related costs. Knowledge of the financial hardship of patients is becoming more and more important for physicians even in a healthcare system with statutory health insurance.

The medical progress in cancer treatment with increasingly new options potentially improving patient outcomes is a challenge for the complete healthcare sector (1) as these achievements in modern medicine are costly and associated with a financial burden for almost every country of the Organization for Economic Co-operation and Development (OECD) over the last years (2). In 2019, health expenditure in Germany was estimated at 410.8 billion euros and made up 11.9% of the gross domestic product (GDP: defined as spending from government and/or social or compulsory insurance funds). After passing the 300 billion mark for the first time in 2012, this rising trend is still ongoing. In 2020, across all 27 European countries, Germany spent the most on healthcare and was almost 2% above the average GDP of all countries (22, 23). In the future, demographic change could lead to even higher costs in Germany and, thus, to an increasing prevalence of financial burden for the society as a whole but also for the affected patient.

In the past, while many studies focused on financial burden in the United States, proving the powerful impact of cancer on the socioeconomic situation and the distinctive consequences, there are fewer studies addressing this issue in Germany and other Western European countries (24). Due to the different types of healthcare systems, there is an undeniable lack of comparability. The USA has a voluntary, private employer-based and individual-based system. Compared with Germany, there is no mandatory enrollment leading to a high proportion of uninsured people (25). However, US citizens over the age of 65 are provided with government health insurance coverage (Medicare). In general, statutory health insurance (SHI) is the primary insurance in Germany for approximately 88% of the population. If the annual income is above the compulsory insurance limit, there is the possibility to choose private health insurance, and exceptions may be self-employed people, who can choose regardless of their annual income. For 2023, the annual limit is 66,600 € per year or 5,550 € per month (26). Although most medical expenditures are covered, co-payments for prescription drugs, hospitalization, and rehabilitation are required, but if a certain limit (2% of the patient’s gross income) is reached within a calendar year, insurees are spared from further payments. This regulation is applied for statutory health insurance because private insurance holders’ deductibles have to be contributed according to the individual insurance policy. Therefore, the finding is not surprising that most HNSCC patients have to face economic deterioration in the form of OOPP and/or loss of income.

The conclusions of this study can only be transferred to a limited extent to other Western European countries as the systems of healthcare finance are different. The predominant systems are public finance by general taxation, public finance based on compulsory social insurance, and private finance based on voluntary insurance. The coverage of medical treatment differs strongly. For example, the coverage of outpatient medical care ranges from below 60% in Italy, Malta, or Portugal to 90% or more in Denmark and Germany, and the coverage for pharmaceuticals is most generous in Germany (82%) and France (81%) (27).

Based on a German cancer patient cohort (*n* = 502), Büttner et al. (28) reported a monthly mean of 206 € in OOPP during the first 3 months after hospitalization and a (*decreased*) monthly mean of 148 € after 15 months, which marks an annual mean of 1,776 € in OOPP. These findings are in line with our results (annual mean of 1,716 € for the financial burden group). However, the dynamic of OOPP over time highlights the importance of continuous monitoring during aftercare. Previous literature focusing on breast cancer suggests that long-term survivors are less affected by OOPP than patients in the initial treatment phase (29). Moreover, OOPPs for different types of cancer are expected to vary; in addition, looking at the international context of OOPP, a greater range of variation can be observed. For instance, Massa et al., who performed a retrospective study in the USA comparing patients with HNC and other cancers, found higher annual median OOPP in HNC ($ 8,384 vs. $ 5,978; difference, $ 2,406; 95% CI $ 795–$ 4,017) and concluded that HNC exacerbates an additional burden to an already financial strained population (9). Furthermore, these differences could be driven by regulations specific to each country, types and extent of healthcare insurances, and social security system (30). Similar to OOPP, the type of cancer has been observed to impact the occurrence and magnitude of income loss. In contrast to the findings of Šaltytė Benth et al. (26), who reported an annual income loss of 3,844 € 5 years after a breast cancer diagnosis, patients experiencing financial burden in our cohort had a higher annual income loss of 7,446 €. In addition, when comparing patients diagnosed with different forms of cancer, individuals with HNC were more frequently found in the lowest income category (3), which means that the relative financial burden of HNC patients is even higher and they may face substantial consequences of financial toxicity that affect QoL. Similar to Mady et al. (31), we found differences by primary site, with the worst financial burden in patients with larynx/hypopharynx cancer, indicating potential site-specific factors, e.g., impossibility to proceed working in the same employment area (for instance, as a teacher) or inability to return to work due to stigmatization or workplace discrimination. Moreover, suffering from HNC and larynx/hypopharynx cancer is associated with long-lasting effects on general health including fatigue, pain, dysphonia, oral dysfunction (trismus, xerostomia, impaired ingestion), and lack of appetite (32–35).

Recent breakthroughs in medical research for HNC treatment, including advancements in surgery and technology, have prevented relapse and have extended the lifespan of patients, decelerated the progression of incurable cancer, and reduced the necessity for harmful and toxic therapies (36). On the other hand, these novel treatments with major surgical procedures, including costs for neck dissection and free-flap reconstruction (37–39) associated with HNC treatment, increased the total healthcare costs (40, 41). Previous studies performed to elucidate the cost of treatment linked to particular treatment modalities reported that trimodal therapy (including surgery and chemoradiation) was the most expensive, whereas surgery alone had the lowest cost, followed by radiation alone, surgery plus radiation, and chemoradiation (39, 42–44). Additionally, these treatments may still lead to significant physical, functional, and psychosocial challenges that can impair patients’ overall functionality (42, 45–48). Nevertheless, we observed no statistically significant differences in association with self-reported financial burden between these treatment modalities. However, patients with reported income loss and/or OOPP, who received surgery and adjuvant therapy and stated to have on average both higher income loss and/or OOPP, need to be considered. This finding proved to be statistically significant when categorizing patients with self-reported financial burden into early and advanced UICC (7th edition, 2017). These particular patients at risk may be additionally overexposed to indirect costs due to long-term side effects of treatment, including lost employment/productivity as well as effects on essential daily and social functions such as swallowing, eating, speech, and communication (49, 50), not to mention being challenged with more frequent hospital visits and potentially seeking more medical interventions.

Interestingly, there was no difference with respect to age (according to age scores) or sex, suggesting that the incidence of financial burden is independent of sex and gender.

In the presented patient cohort, out-of-pocket costs were quantitatively more prevalent than income loss. The majority of patients (50%) had to spend money that was not reimbursed by their insurance. Half of the affected patients stated that they had to pay for traveling without compensation, as shown in **Table 2**. As the mean age of our cohort is 65 years, it has to be considered that many patients may rely on public transportation or relatives to drive them to their appointments, which may cause additional everyday stress. However, it is unknown which amount of money this proportion takes compared with overall expenditures. The results from the study of Baili et al. showed a significant difference in OOPP due to travel to the hospital and/or cancer specialist, depending on where the patients lived in Italy (7). Further multicentered research in Germany is needed to address this question, providing an overview of the amount and infrastructure and the importance of seeking healthcare at specialized cancer centers, even though these are far from the residence of patients. Providing information about the living situation (urban or rural), which could possibly have influenced the amount of OOPP, because the quantitative proportion of transportation costs was significant, should be routinely assessed and considered in further studies to show the possible impact of new healthcare reforms. The trend to allocate complex treatments to certain hospitals while the number of services a single clinic offers will likely decrease goes in hand with further traveling for the patients (51). This may burden patients living in rural areas more and needs to be acknowledged (52–54).

Additional payments in SHI are mainly for medical supplies and dentures, so it is not surprising that 52% of the financial burden group suffered from co-payments. In general, dental prostheses were only reimbursed for SHI beneficiaries to half of the costs. A cross-sectional study using data of *n* = 3,124 subjects aged 57 to 84 years from a population-based prospective cohort study [ESTHER study (2)] collected from 2008 to 2010 in Saarland, Germany, examined inequalities in OOPP among elderly Germans. Bock et al. were able to highlight the mean OOPP of 119 € per capita during a 3-month time period, of which 34% was spent on medical supplies, 22% on dentures, and 21% on pharmaceuticals (2). Aside from their low sociodemographic and association with insufficient dental hygiene, patients with HNC are particularly at risk for requiring a dental prosthesis after therapy as the treatment of HNC may include oral surgery and/or the extraction of teeth or loss due to radiotherapy.

**Figure 3:**
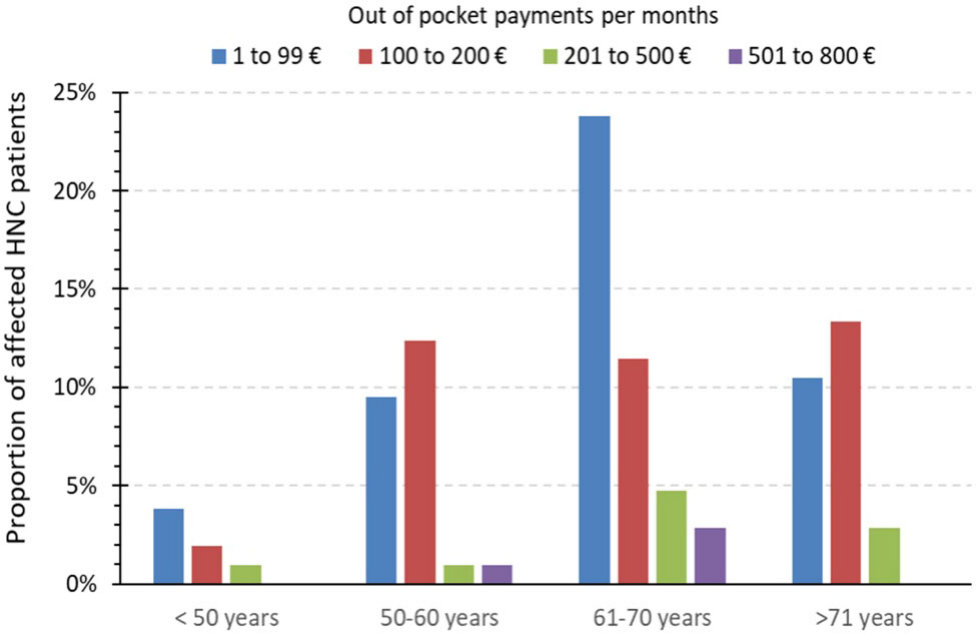
Extent of monthly out-of-pocket payments in the financial burden group. Bar indicating the amount of monthly out-of-pocket payments (in percent) of all affected patient with OOPP (*n* = 100) in comparison to different age groups.

A positive association between increasing age and higher OOPP was not in line with our findings. Due to the fact that the mean age of these cohorts was only distinct by 5 years (Bock et al.’s mean age of 69 years vs. our mean age of 64 years), we assume that a cancer disease, regardless of the time of diagnosis, has such high impact on patients that we could not see differences in the age scores. Younger patients who should potentially be still able to work are more impaired from disease-related consequences because they may suffer more due to income loss, and older/retired patients may suffer more due to OOPP (**Figure 3**). Unemployment due to cancer disease was a significant factor for financial burden (OR 5.423, 95% CI 1.501–19.59).

Mehlis et al. who investigated the financial burden of colorectal (*n* = 125) and neuroendocrine (*n* = 122) stage IV cancer patients at a German Comprehensive Cancer Center showed that 81% of the patients were affected by OOPP and 37% faced income loss as a consequence of their disease (20). However, we would agree with their interpretation that income loss outweighs OOPP because the reported payments in our study did not exceed 200 € monthly in 80% of the cases, while almost one-third of the affected patients reported more than 800 € monthly income loss. If our cohort also included only stage IV cancer patients, we may have seen even higher results, based on the fact that 75% of patients experiencing financial burden suffered from advanced disease.

The high incidence and mortality of HNSCC are often linked to a lifestyle characterized by alcohol consumption and tobacco smoking, which are well-known risk factors for HNSCC and other cancers. Patients with HNC are more often less educated, poorer, sicker, and lacking private insurance. Also, medical and out-of-pocket expenses are higher for HNC patients than for other cancer patients (9). In our cohort, most of the patients also had a low level of education. Collectively, these results may lead to disfranchisement regarding the socioeconomic situation, impaired QoL, and worse outcome of therapy, considering that patients experiencing financial toxicity are more likely to have lower medical compliance and skip clinic visits (55). An online survey conducted in the USA in 2018 showed that cancer patients are more worried about financial depletion and its consequences than dying of cancer (56). We believe financial toxicity may be a barrier to the best possible cancer care and, hence, may impact patients’ decision-making and future treatment choices ultimately, which is negatively influencing their overall survival.

It is worth noting that our results, suggesting self-reported financial burden, and its impact on QoL, are important in the German public health system. We would like to propose the following perspectives for healthcare, policymakers, and the next generation of research.

Offering cancer patients support concerning financial hardship should be considered. The future of a multidisciplinary team should not only consist of doctors, psycho-oncologists, nutrition consultants, and other professionals but also integrate financial advisors for consecutive assistance. To target vulnerable patients, an implemented screening tool in cancer medical care is needed. There is an unmet need for a (implementation of a) standardized instrument in the context of publicly financed health systems in Germany or Europe, and hence, more research needs to be conducted to develop a tool indicating high-risk groups. We recognize that there have been increasing efforts, on a global level, aiming at this subject. For example, de Souza et al. designed an 11-item tool (COST score) to quantitatively measure cancer patients’ experiences related to financial distress (57). The comprehensiveness of this tool would make it easy to implement in the clinical visit routine. It is well established that a longer estimated time to complete a questionnaire is linked to a higher non-participating rate; therefore, we assume this tool could achieve a better tolerance and completion rate in short-term visits. However, due to differences in the healthcare system, it is not suitable to transfer it in our context. Nevertheless, it is necessary to acknowledge the financial hardship of HNSCC patients and actively provide information for politicians and policymakers on how to improve their situation and develop new guidelines. In the future, there will be new challenges, such as increasing medical costs, rising incidence, and demographic changes.

Our study has some limitations that need to be discussed. Given the character of this study that patients were not asked multiple times during their cancer survivorship, information about the dynamic of financial burden during the course of the disease and its treatment is missing. We assume that a longitudinal study could help fill the gap of knowledge about changes in the amount of expenditures. A short time since diagnosis may not be sufficient to capture the financial consequences of cancer, which may take some time to emerge fully. Those who have survived a long time post‐treatment may have experienced hardship in the past, but that could have resolved by the time of questionnaire completion, so it is possible that we have underestimated the true prevalence of financial toxicity. Furthermore, it has to be mentioned that there is no standardized questionnaire to address financial burden for all cancer patients, so the comparability of study results is still unsatisfying. We suggest the development and implementation of a standardized screening tool for all cancer aftercare programs, which should be used at the time of diagnosis and also during aftercare visits to identify patients who need more assistance.

Furthermore, non-respondents were not recorded; therefore, socioeconomic status and financial burden between survey respondents and non-respondents could not be compared. We assume that the group of non-respondents is characterized as less educated with a lower socioeconomic status and, therefore, could face hardship, but do not seek any support nor attention concerning their current situation, which could underestimate our findings. In the interest of this unverified hypothesis, we suggest that in further study settings, non-respondents may be given the option to state their reason. Also, we did not ask our patients if they desired support in the form of assignment to a financial advisor when they faced financial burden. Our paper has several limitations. However, being able to show the prevalence of financial burden and comprehensive information on the socioeconomic and clinical characteristics of HNC survivors is among the strengths of this study. The patients represent a broad socioeconomic cross-section of German HNSCC patients, and therefore, conclusions from this study can be applied to German HNSCC patients broadly. Lastly, the analysis makes use of a large sample size with different HNC entities from the cancer aftercare program within the Department of Otorhinolaryngology of a large university hospital in Germany, which identified the subgroups of locally advanced tumors and advanced larynx/hypopharynx having the highest risk for significant financial burden.

## Conclusion

The present study demonstrates a substantial socioeconomic impact of HNC and especially local advanced HNSCC on patients even in the German healthcare system with statutory health insurance and shows that German HNSCC patients suffer from significant financial burden. In the context of improved overall survival and increasing cancer care costs, this phenomenon becomes even more relevant in the future for healthcare workers, the healthcare system, and their decision-makers. Modern HNSCC aftercare programs must implement standardized screening tools for financial burden and should routinely assess it, even before the initiation of therapy, to identify patients who are at risk for financial toxicity and related incompliance in clinical aftercare increasing their risk for and/or delayed detection of relapse. Further research is needed to investigate predictors, to what extent, and at which point of time HNSCC patients face (*high*) financial burden and experience deterioration of quality of life and which activities raise the acceptance of psycho-oncological or financial support in these patients. Financial advisors, psychologists, and patient navigators should be considered as essential team members in multidisciplinary HNSCC care and should be contacted at least once during the cancer survivorship. To address financial hardship, financial advisors can give educational sessions to increase awareness of available financial assistance resources and help with applying for health insurance coverage and assistance programs like the German Cancer Aid fund for cancer patients in financial need. Moreover, additional support programs are needed to help establish systematic methods to enhance financial navigation services.

Understanding the prevalence and consequences of financial toxicity may help to identify the needed modification of future head and neck oncology programs and possible improvements of an enhanced patient experience and outcome.

## Data availability statement

The original contributions presented in the study are included in the article/supplementary material. Further inquiries can be directed to the corresponding author.

## Ethics statement

The studies involving humans were approved by Ethics Committee of the Medical Faculty of the University Leipzig (vote 289722-ek). The studies were conducted in accordance with the local legislation and institutional requirements. The participants provided their written informed consent to participate in this study.

## Author contributions

JR: Writing – original draft, Formal Analysis, Investigation, Methodology, Validation, Writing – review & editing, Data curation. VZ: Investigation, Writing – review & editing. AD: Investigation, Writing – review & editing. GW: Data curation, Formal Analysis, Investigation, Methodology, Validation, Writing – original draft, Writing – review & editing. SW: Formal Analysis, Investigation, Methodology, Validation, Writing – original draft, Writing – review & editing, Conceptualization, Supervision.
